# Variable plate kinematics promotes changes in back-arc deformation regime along the north-eastern Eurasia plate boundary

**DOI:** 10.1038/s41598-024-57890-6

**Published:** 2024-03-27

**Authors:** Eleonora Ficini, Marco Cuffaro, Carlo Doglioni, Taras Gerya

**Affiliations:** 1 CNR-IGAG, Rome, Italy; 2 National Institute of Geophysics and Volcanology, Rome, Italy; 3 Department of Earth Sciences, Sapienza University of Rome, Rome, Italy; 4 Institute of Geophysics, ETH Zurich, Zurich, Switzerland

**Keywords:** Solid Earth sciences, Geodynamics, Geophysics

## Abstract

The stretching of the lithosphere leading to back-arc basins formation generally develops behind arc-trench systems and is considered the consequence of slab retreat relative to the upper plate. Here, we examine the deformation regime evolution within the overriding plate due to subduction processes, using thermo-mechanical numerical simulations. We explore the north-eastern Eurasia plate boundary and the mechanisms of subducting Pacific plate since 57 Ma. During this time interval, several extensional basins formed along the Eurasia margin, such as the East China Sea, the Japan Sea, and the Kuril basin. Here, we increased the simulation complexity, with the inclusion of (i) the kinematic variability of the Pacific plate over the geological past with respect to a fixed Eurasia, incorporating time-dependent (i.e., temporally evolving) velocities computed from plate motion reconstructions; (ii) a Low-Velocity Zone within the asthenosphere, and (iii) a horizontal eastward mantle flow. Our results show a crucial role of the mantle flow for the development of lithospheric extension and back-arc basin opening, and a main kinematic control of the subduction trench position, which advances and retreats, into distance intervals in the order of $\sim$ 100 km, and providing stages of compression and extension in a back-arc basin.

## Introduction

There are several assumptions at the base of back-arc basins formation, which can be the results of different processes, such as mantle motion, or the retreat dynamics, respectively^1–4^. However, interactions among a subducting slab, the upper plate, and the mantle govern their occurrence, whether within oceanic or continental plates. This is confirmed by geological and geophysical evidence, such as the timing at which most of the back-arc basins developed and began their closing stage in north-eastern Eurasia^5–10^, which suggests a common trigger, i.e., events related to subduction of oceanic lithosphere^4^. Nonetheless, the subducting plate velocities play a role both in back-arc basin evolution, and slab morphology and dynamics. Plate kinematic variations are not considered in most of numerical models, which assume constant plate velocities rather than incorporating reconstructed time-dependent velocities, that are indeed expected to impact the slab behavior^11–13^.

Back-arc basins are mostly located throughout the entire western Pacific margin, whereas they are almost absent on its eastern rim^5,14^. Their opening occurred, and in some cases it still does, mainly along subduction zones that have ’westward’-directed slabs^15–17^, so that a relation between slab dynamics and the divergence of the subduction hinge with respect to the upper plate is suggested^14,18^. This global geodynamics implies the action of a horizontal ’easterly’-directed mantle flow^19–22^. However, compression, associated with large-scale changes in the tectonic regime, may inhibit extension and thus the opening of a back-arc basin^23^.

**Figure 1:**
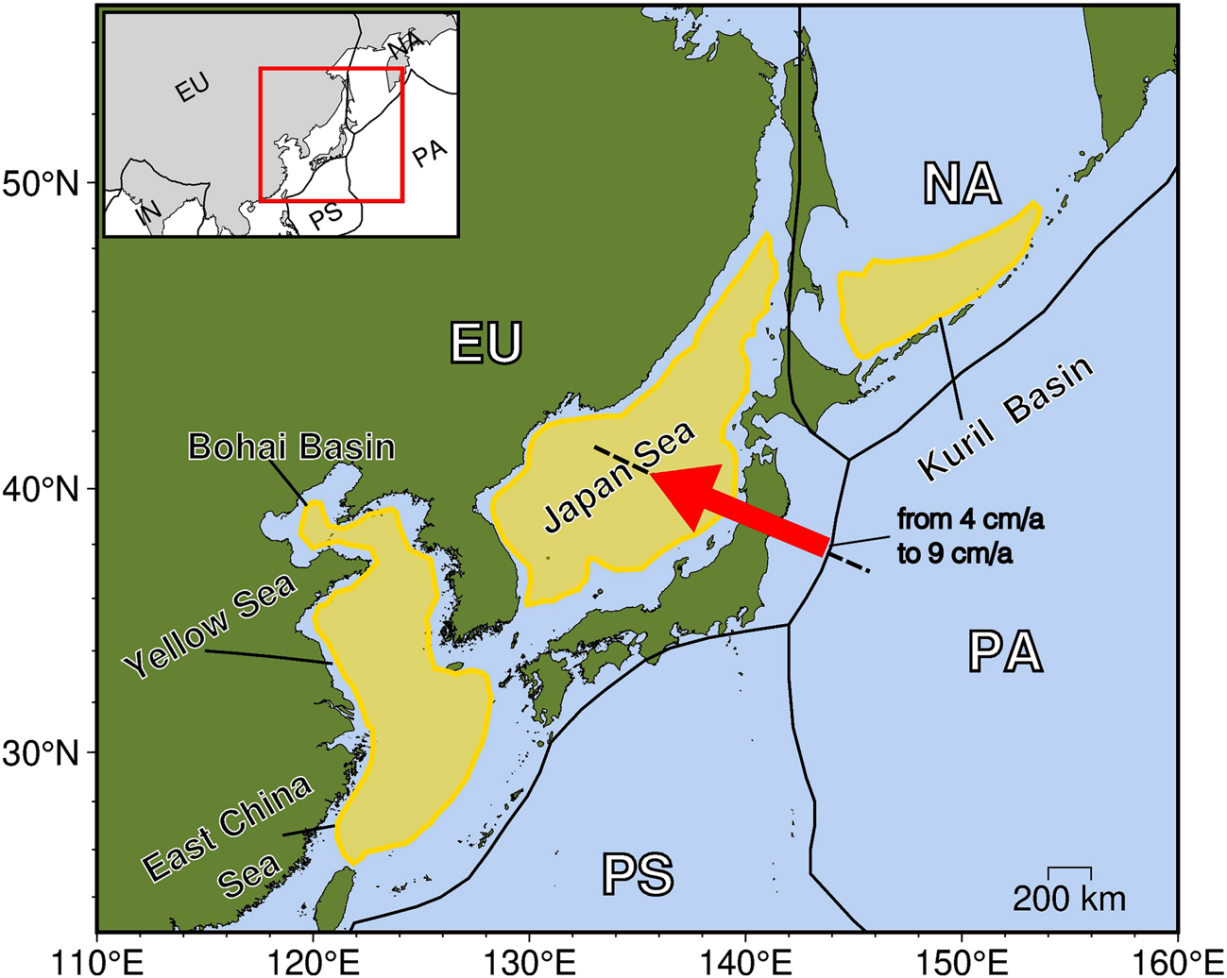
North-eastern Eurasia plate boundary. In this study area back-arc basins were interested by alternated extension and compression in time during the Cenozoic. Black dashed line represents the profile along which the reconstructed Pacific plate velocities (red arrow) with respect to a fixed Eurasia are computed. This figure was generated using PyGMT^56^.

The recent subduction history of the Pacific plate under the Eurasia continent begins in the Late Paleocene/Early Eocene, when the subduction of the Izanagi-Pacific spreading ridge occurred. Then, around 57 Ma, a very young Pacific lithosphere started to subduct below the Eurasia continent showing different plate velocities^24^. Several basins developed during the Cenozoic (Fig. 1), such as the Kuril Basin, the Japan Sea, the East China Sea Shelf, the Yellow Sea, and the Bohai Basin, that were interested by phases of both extension and compression^25–28^. For instance, the Japan Sea and the Kuril Basin provide current evolving examples of back-arc basins which opened within a continental plate during the Late Eocene-Miocene. They both should be presently in their closing stage, in Japan after going through a stage of neutral stress regime, whereas in the Kuril area after a stagnation period^29–33^. Southward, the East China Sea Shelf, the Yellow Sea, and the Bohai Basin went through extension with some phases of compression^25–27,34^.

**Figure 2:**
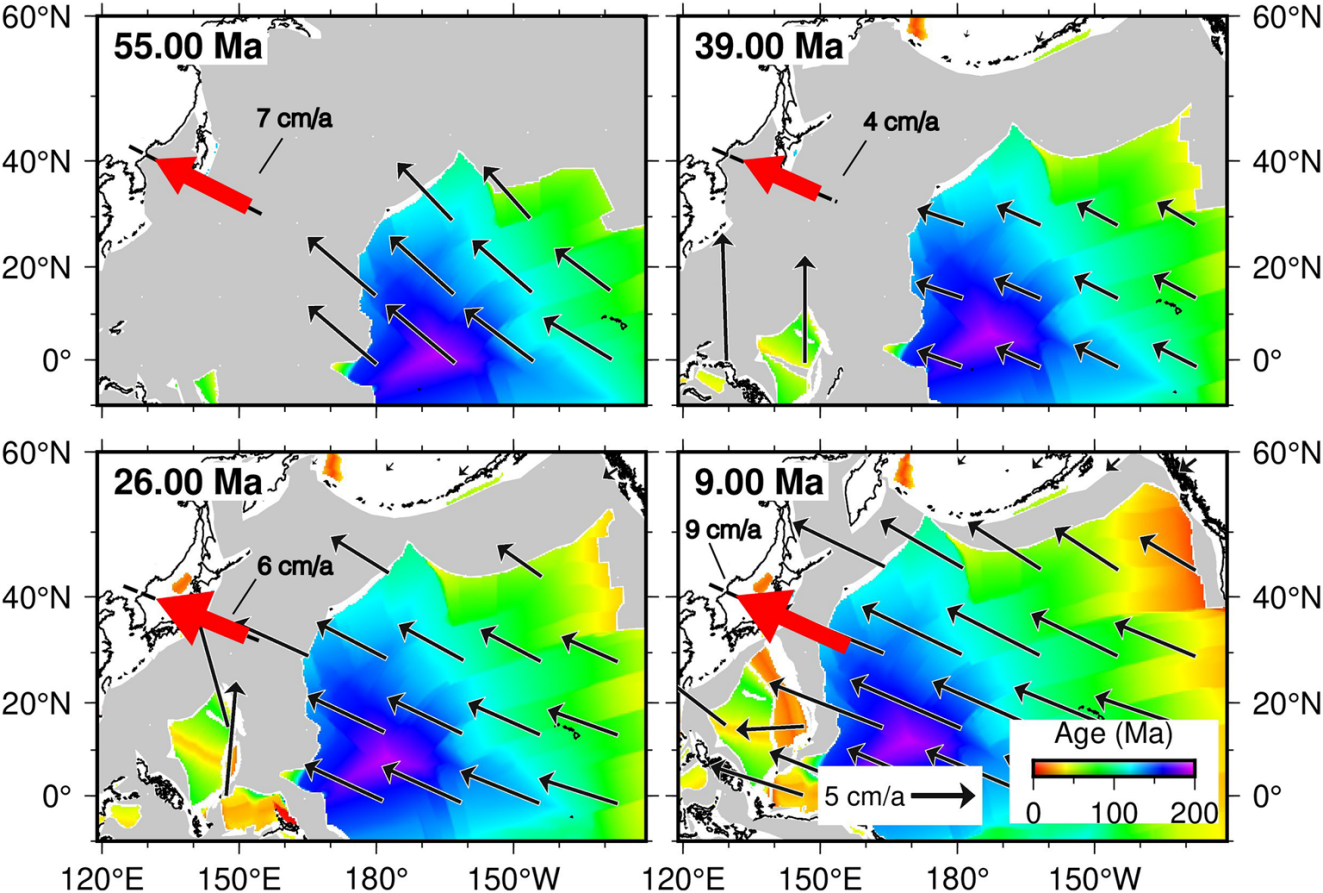
Pacific plate motion reconstructions. Reconstructions were computed with respect to a fixed Eurasia using updated finite rotations by^24^, with the inclusion of corrections to the Pacific displacements prior to 83 Ma^45,57^ (black arrows). In this figure, the colors represent the age and shape of the current Pacific oceanic lithosphere, whereas the gray areas are the oceanic lithospheric plate subducted until nowadays. Black dashed lines show the profile along which the Pacific plate velocities (red arrows not in scale) are computed.

However, the Pacific plate motion variability (Fig. 2, Tab. S1 Supplementary Information) and its subduction processes beneath Eurasia, gave the main contribution to the geodynamic evolution^4,16,33,35–44^. Thus, in this paper we provide 2D numerical simulations of the interactions between the Pacific and north-eastern Eurasia plates during the last 57 Ma, to investigate the role of variable plate kinematics of a subducting plate on the evolution of a subduction zone and the relative deformation regime along the continental upper plate. Our models include the Pacific plate motion changes^45^, computed along the profile reported in Figures 1 and 2, a Low-Velocity Zone (LVZ^14,18,22,46–53^), and the mantle flow^22^. This latter is also based on geochemical evidence in the composition of Cenozoic basalts in the area, revealing an eastward shift of the intraplate and arc volcanism through time during the Eocene-Early Miocene^28^, so that an eastward mantle flow needs to be addressed as the primary cause of this eastward shift. Furthermore, our approach is similar to the one by^22^, in which the westward lithospheric drift (or relative eastward mantle wind) is included in the model^54,55^.

In this paper, we carried out two models to methodologically describe these processes, both using velocity reconstructions of the Pacific plate with respect to fixed Eurasia in the last 57 Ma^24^: Model 1, without the inclusion of mantle flow, and Model 2, with the use of mantle flow. The 2D profile where the Pacific plate motion history is reconstructed is located at the Pacific-Eurasia plate boundary, in the place where the two plates have been continuously in contact since 57 Ma until today (Figs. 1 and 2).

## Results

### Model 1 (without mantle flow)

**Figure 3:**
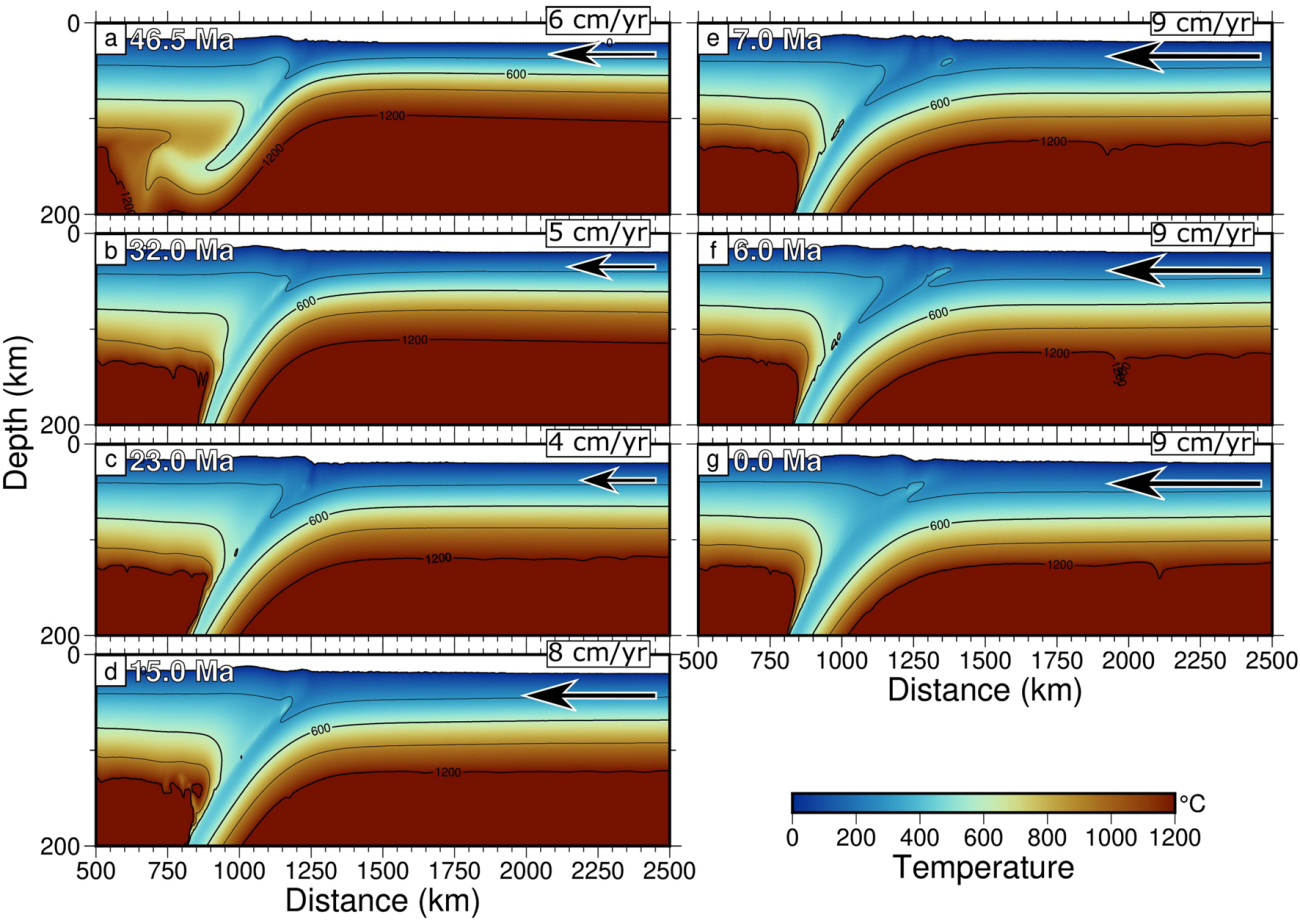
Results of model with LVZ and no mantle flow for the subducting Pacific plate under Eurasia since 57 Ma. The shallower portion of the slab (200 km depth) shows an almost constant dip during the entire 57 Ma evolution of the model. Here, no back-arc basin is opening.

Results of Model 1, without mantle flow (Fig. 3, Fig. S2, and Supplementary Movie SM1 in the Supplementary Information), show no back-arc basin opening throughout the entire subduction evolution, with a slab denoting an almost constant dip. Model 1 also provides an overall small trench retreat and advance, in the range of 100 km (Figs. 3 and 5b, Fig. S2 and Supplementary Movie SM1 in the Supplementary Information). In Model 1, this occurs regardless of the Pacific plate velocity changes through time. At depths (Fig. S2 Supplementary Information), within the wedge in front of the subducting slab, no mantle counterflow is detected, able to induce slab retreat and eventually leading to back-arc basin opening.

### Model 2 (with mantle flow)

**Figure 4:**
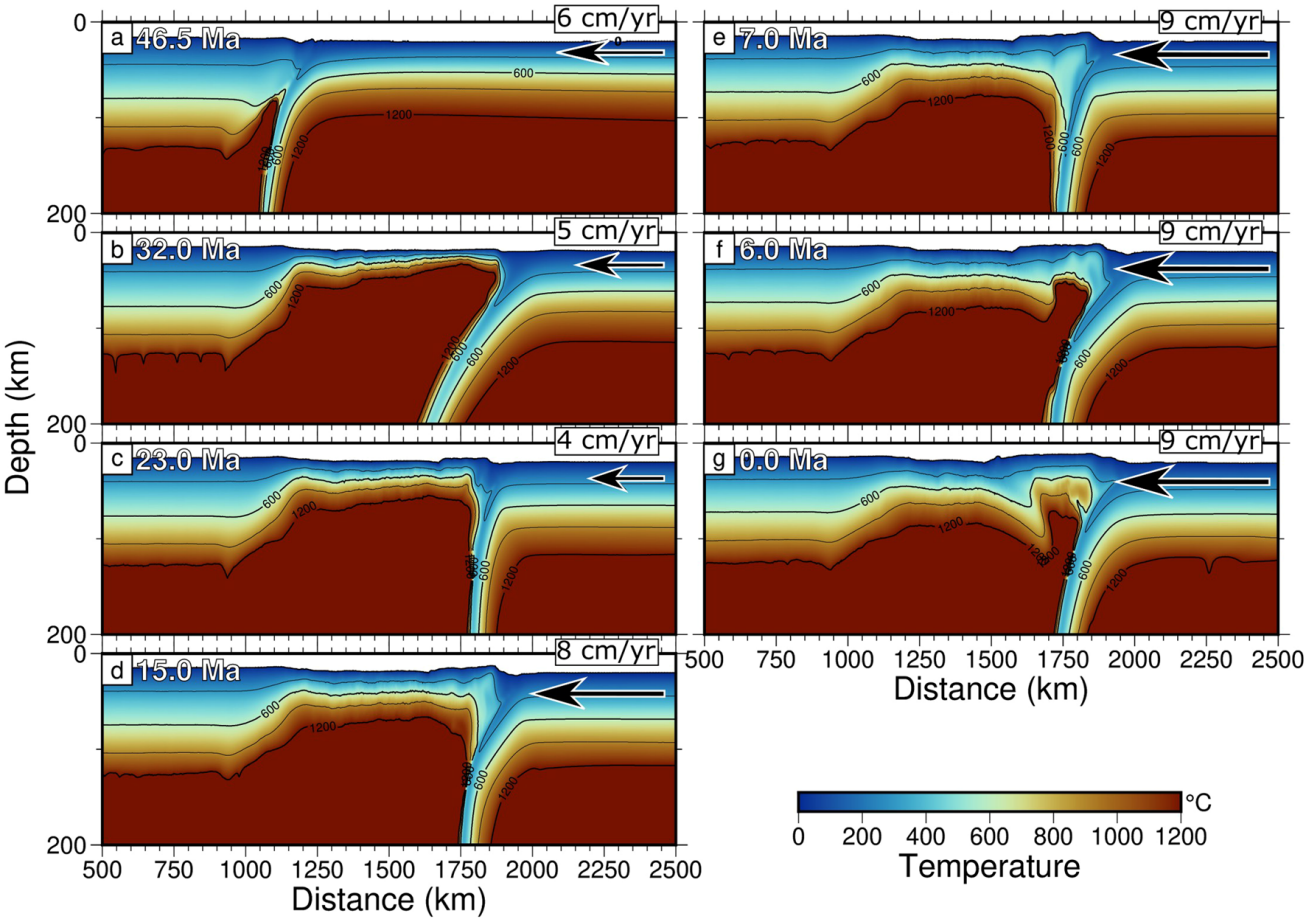
Results of model with LVZ and mantle flow for the subducting Pacific plate under Eurasia since 57 Ma. An extensional phase can be detected, beginning at $\sim$ 46.5 Ma (panel **a**), with a developed back-arc already at $\sim$ 32 Ma (panel **b**). Then, a compressional phase starts (panel **c**), ended at $\sim$ 23 Ma, when a new extensional phase begins. At $\sim$ 15 Ma (panel **d**) a new inversion of the extensional trend starts. A very short opening trend is again active from $\sim$ 7 Ma (panel **e**), whereas at $\sim$ 6 Ma a new inversion develops (panel **f**). Panel g shows the evolution at 0.0 Ma (Present-day), which consists of a compressional stage.

**Figure 5:**
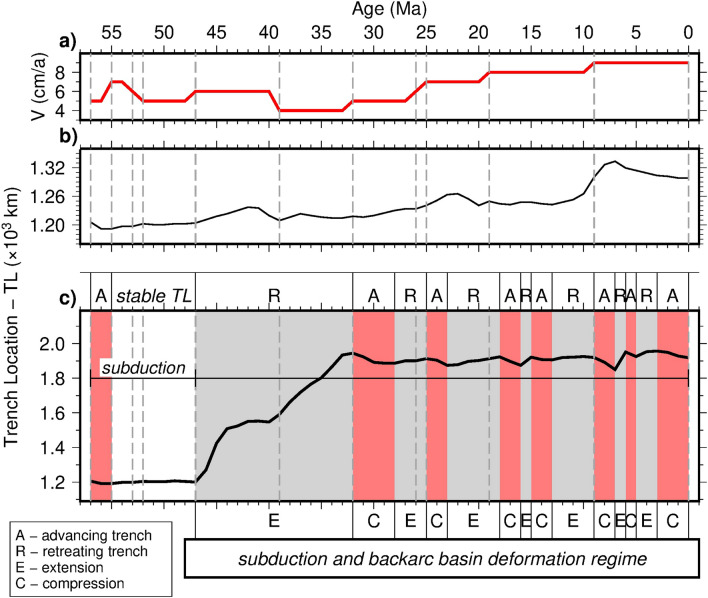
Deformation regime and trench motions. (**a**) Changes of Pacific plate velocity with respect to fixed Eurasia^24,45^. (**b**) Trench location (TL) for simulations with variable plate kinematics, LVZ and without mantle flow. (**c**) Trench location for simulations with variable plate kinematics, LVZ and mantle flow. During the subduction process, without the inclusion of the mantle flow (**b**), short retreating trench phases only correspond to change in plate kinematics, and back-arc basin does not form. On the contrary, with the inclusion of mantle flow (**c**) we observe an initial trench advance (light red sectors) and a stable trench location from 57 to 47 Ma. After that back-arc basin forms with seven advancing trench and compression phases (light red sectors), and seven retreating and extension ones (light gray sectors), as the result of the interaction between Pacific plate motion changes and horizontal mantle flow.

Results of this numerical simulation (Figs. 4 and 5c, Fig. S3 and Supplementary Movie SM2 in the Supplementary Information) show that, after a short initial advancing trench location from 57 to 55 Ma, the trench is mostly stable from 55 to $\sim$ 47 Ma (Fig. 4b, c, and Supplementary Movie SM2). During this time, plate velocities change back and forth from 5 to 7 cm/a (Fig. 4a). After that, at $\sim$ 47 Ma the slab interacts with mantle flow and the extensional phase in the back-arc basin begins (Fig. 4a), with a developed back-arc already at $\sim$ 32 Ma (Fig. 4b). This is well shown by the analysis of the trench location (Fig. 5c), where it moves away from the upper plate, going from $\sim$ 1200 to $\sim$ 1944 km. At this stage, the resulting thin lithosphere in the back-arc basin ($\sim$ 10 km) locates at $\sim$ 1750 km (Fig. 4b).

At 32 Ma, a short compressional phase starts, ending at $\sim$ 28 Ma. At this stage, extension begins again, ending at 25 Ma, whereas compression develops until 23 Ma (Fig. 4c). Velocity slows down during this period from 6 (47 Ma) to 4 cm/a (33 Ma), to increase again until 7 cm/a (25 Ma) and, during this range of time, the trench moves respectively from $\sim$ 1944 to $\sim$ 1886 km, then from 1886 to 1913 km, and finally from 1913 to 1874 km, relative to the upper plate (Fig. 4c). At $\sim$ 23 Ma, a new extensional phase begins, until $\sim$ 18 Ma. Then a short compressional phase occurs until 16 Ma, and a further very short extensional phase is emplaced until 15 Ma (Fig. 4d). During this time, the trench moved respectively from $\sim$ 1874 to $\sim$ 1923 km, then to $\sim$ 1874 km again, to shift at $\sim$ 1921 km at the end of the last extensional phase (Figs. 4d and 5c). At this last stage, a short new inversion of the extensional trend starts, until $\sim$ 13 Ma, when a new extensional phase emplaces until 10 Ma (trench at $\sim$ 1925 km). During this time, velocities change from 7 to 8 cm/a.

A compressional phase, which makes the trench jump from $\sim$ 1925 to $\sim$ 1850 km, starts from $\sim$ 10, to $\sim$ 7 Ma (Fig. 4e), with an extensional impulse occurred between 7 and 6 Ma, with the trench jumping from $\sim$ 1849 to 1951 km (Fig. 4f). A 1 Ma compression occurs again between 6 and 5 Ma, with the trench going from $\sim$ 1951 to 1925 km. During this compressional phase, the oceanic lithosphere thickened ($\sim$ 50–60 km) at the left side of the subduction, in the back-arc basin, and starts to subduct with a slab dipping towards the east, opposite to the primary subduction direction (Fig. 4e). A short extension occurs between 5 and 3 Ma and, during this last extensional event, the trench jumps from $\sim$ 1925 to $\sim$ 1956 km. After this, compression emplaces until Present-day (0 Ma), and the trench moves from $\sim$ 1957 to 1918 km (Fig. 4g).

Eventually, in our model, the final width of the basin appears to be of approximately $\sim$ 714 km which roughly corresponds to the maximum extension occurred along the north-eastern Eurasia margin (Fig. 1). It is worth noting that from 32 Ma, age at which we observe a minimum lithospheric thickness ($\sim$ 10 km) and maximum extension within the back-arc basin, the lithosphere starts to thicken again, reaching its maximum of $\sim$ 50 km (Fig. 4g). Extension in the upper plate lithosphere occurs for about 150 km throughout the entire model evolution. From $\sim$ 1050 km, where the continental plate boundary is located at the beginning of the model run, to $\sim$ 1200 km at its end. This is evident in Fig. S3 (Supplementary Information).

These model results at depths (Fig. S3 and Supplementary Movie SM2, Supplementary Information) show that the slab is entirely influenced by the eastward mantle flow in the initial opening phases of the back-arc basin, whereas it is mainly influenced by the mantle flow below 200 km in the subsequent phases. In fact, within the first 200 km depth of the numerical domain, where the LVZ decollement level is located, the velocity field has no unique direction and shows, in the subduction wedge, several phases of alternating westward and eastward directions. Moreover, a slab stagnation is evident at about the 660 km discontinuity (Fig. S3 and Supplementary Movie SM2, Supplementary Information).

## Discussion

In the last 57 Ma, our numerical modelling results highlight different subduction trench displacements during the evolution of the Pacific subduction along the north-eastern side of the Eurasia plate (Fig. 5). In Figure 5, the deformation regimes and trench motions of the two models, carried out within this study, are reported: Model 1 (panel 5b), without mantle flow, and Model 2 (panel 5c), with mantle flow, whereas in panel 5a, changes of Pacific plate velocity with respect to fixed Eurasia are provided. Simulations with variable plate kinematics and without mantle flow show a small ($\sim$ 33 km) trench retreat, boosted by every plate velocity change (Fig. 5a and b) without back-arc basin opening (supported by Fig. 3, and Fig. S2 and Supplementary Movie S1 in the Supplementary Information). On the contrary, simulations with variable plate kinematics and mantle flow present the opening of a well-developed back-arc basin (Figs. 4 and 5c, Fig. S3 and Supplementary Movie SM2), with seven retreating phases and seven advancing ones. The alternating behavior of the trench (i.e., advancing, retreating but also neutral) is documented by numerical models in literature^58,59^ and it is mostly related to some spontaneous changes in plate coupling due to mantle melting, which is not included in our computations, or to changes in the age of the oceanic plate and of the constant convergence rate.

In our models, this behavior is strictly related to subducting plate velocity changes through time^41^. In fact, a general correspondence between the ages at which velocity variations occur (Fig. 5a and Supplementary Tab. S1) and tectonic deformation changes from predominant extension to predominant contraction in the basin (Figs. 4, 5c, and S4 in the Supplementary Information), is here obtained, with trench displacement in the order of $\sim$ 2 to $\sim$ 150 km (Fig. 5c).

Model 2 (Figs. 4 and 5c show a first main extensional phase during which almost the entire back-arc basin opening occurred, between 47 and 32 Ma, producing a general trench retreat of $\sim$ 740 km (Figs. 4 and 5c, Fig. S3 and Supplementary Movie SM2 in the Supplementary Information). In this time span of $\sim$ 15 Ma the lithosphere within the basin reached the minimum in thickness ($\sim$ 10 km). This is in accordance with^28^, that pointed how the eastward motion of the mantle flow in the area resulted in the first eastward shift of the back-arc extensions in north-east Asia between $\sim$ 52 Ma and $\sim$ 33 Ma. This main opening phase of the basin coincides with the only episode of lowering of the Pacific plate velocity in our model. This is evident in Figure 5, comparing panel 5a and panel 5c, and from Supplementary Table S1, where velocity changes are described.

Then, from $\sim$ 32 Ma, the pure extension within the basin ceased, and extension and compression became competing processes, with increasingly predominance of compression towards the present (Figs. 4, 5a,c, and S4 and Supplementary Movie SM2 in the Supplementary Information). This phase occurs together with a continuous increase in the Pacific plate velocity in time (comparison between Fig. 5a and c, Supplementary Table S1 in the Supplementary Information). This behavior was denoted also by^60^, although^61^ recently described an extension phase going from $\sim$ 15 to $\sim$ 13.5 Ma, as also obtained by numerical simulation in this study (Figs. 4c and 5c).

Looking at our Model 2 at depths (Fig. S3, Supplementary Information), within the wedge zone, phases in which the circulation is towards the west can be observed above 200 km, in accordance to what shown in^62^, where the LVZ lubricates and allows the relative motion between the lithosphere and the asthenospheric mantle. Below 200 km depth the eastward mantle flow predominates. However, the presence of an eastward mantle flow is strictly necessary for the opening of the back-arc basin (Figs. 4 and 5c).

It is important noticing that our resulting slabs below 200 km depth in our models should be all verified using mantle tomography, starting with a robust analysis on the reference model used for its computation and method limitations^63^. We consider, thus, this further analysis out of the aim of this work, since it focuses mostly on the deformation within the back-arc basin in response to plate kinematics, with evaluation of the trench motion.

Episodic extensional and contractional tectonics in the Eastern side of the Eurasian continent are well documented during the entire Mesozoic and Cenozoic^26,64–66^. Indeed, compressional phases in the Late Eocene, Early Miocene, and Late Miocene are observed, in agreement with phases shown by our model results. At $\sim$ 3.5 Ma the compressive stress regime prevailed^28,30,32,33,67^. For instance, as a result of this compression, an incipient subduction is inferred by stress, seismicity, and seismic lines on the western side of Japan, currently involving Eurasia and N-America plates^4,5,68^. Evidence of a current compression is also revealed by recent seismicity, such as the January 1st 2024 Noto Peninsula earthquake (Mw7.5), with reactivation of normal into reverse faults^69–73^. In our model, an incipient subduction is also induced on the western side of the wedge, during the last contractional phases (Fig. 4f and g). It occurs because of compressional stresses acting on a preexisting weakened layer (Figs. S3 and S5, and Supplementary Movie SM2, in the Supplementary Information) within the thickened lithosphere ($\sim$ 30–50 km^74^), emplaced at the end of the main extensional phase (e.g., Fig. 4b, and 7–6 Ma in the Supplementary Video SM2). According to^75^ these incipient subductions appear to be often strictly related with changes in regional kinematics, at which, in fact, ridges and former subductions represent the lithospheric weaknesses where subduction initiation is originated. Similar behavior has been observed in^76^, where, along a spreading center, a new subduction can occur as a consequence of arising compressional regimes.

### East China sea

**Figure 6:**
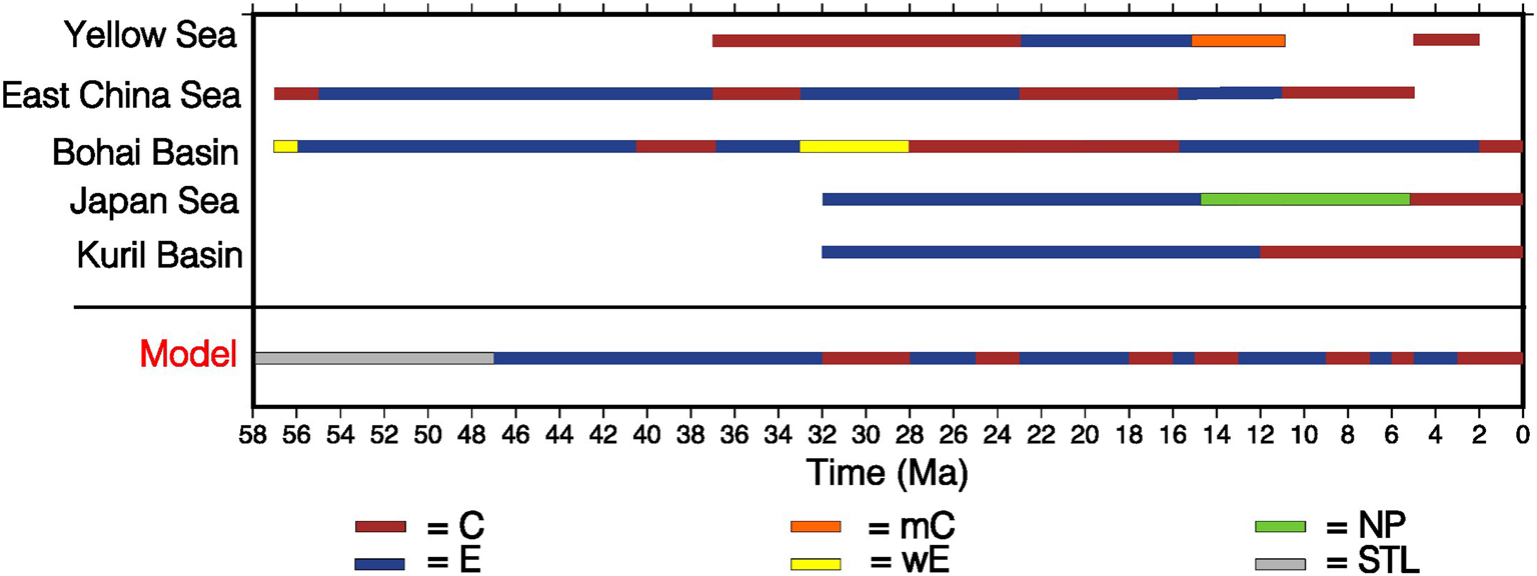
Tectonic regime for the Eurasia upper plate compared with model results. In this figure a comparison between the deformation and tectonic regime observed from geological, geochemical, and geophysical data collected from literature. Red color is used for compressional phases (C), whereas blue is used for extension (E). Orange color is for mild contraction (mC) in the Yellow Sea described by^79^. Yellow color is for wakening of extension (wE) for Bohai Bay Basin as in^34^. Green color is for the neutral phase (NP) of alternating compression and extension described by^28,33,67^ in Japan Sea. Light grey is for stable trench location in the model. Blanks are for no information retrieved. References: Yellow Sea^27,78,79^; East China Shelf Basin^26^; Bohai Bay Basin^25,34^; Japan Sea^23,28,31,33,61,81^; Kuril Basin^29,30,32,36^.

In Eastern China, extension can be most likely related to the subduction of the Pacific plate. In fact, timing of extension and compression within the back-arc basin correlates well with the convergence rate of the subducting plate^34,37,77^. This kinematic control on back-arc basin formation in East Asia is observed comparing the rate of Pacific-Eurasia convergence with the average strain rate in the Bohai Bay Basin during the Cenozoic^34^. In this basin, the peak of extensional stress occurred during the Middle-to-Late Eocene, when the Pacific-Eurasia convergence rate was at a minimum (Fig. 6). This relation inversely correlates the stretching within the Bohai Bay Basin to the relative motion rate of the Pacific plate with respect to the Eurasia plate^34^. The stretching slowed down at about 32–20 Ma, coinciding with the end of the main extensional phase of the back-arc basin in our model, whereas weak compression is inferred at 2–0 Ma^34^ (Fig. 6). In^25^ compression events are registered at 40 Ma and between 28–16 Ma, in this same area (Fig. 6).

Moving south-eastward in this area, also the Yellow Sea underwent through phases of pulsation between compression and extension, since its evolution beginning $\sim$ 250 Ma^78^. During the Cenozoic this area experimented tectonic inversion from extension to compression during Late Eocene-Oligocene, and Pliocene^27,78,79^ (Fig. 6).

The East China Sea Shelf Basin formed depocenters which migrated from west to east, due to the subducting Pacific plate velocity reduction, which enhanced trench retreat^26^. During its evolution history, it has experienced strong tectonic extension and four phases of compressive inversions which occurred in the Late Paleocene, Late Eocene, Early Miocene, and Late Miocene^26^ (Fig. 6). In the Bohai Bay Basin, a similar evolution occurred^25,26^ and, in this area compression events seem to be related to changes in the Pacific plate kinematics and to interactions with the Philippine Sea plate, the Ryukyu Arc, and the Luzon Arc^80^.

### Japan basin

In our model, the main opening phase within the basin started at about 47 Ma and ceased at about 32 Ma. This latter is the time at which geological and geophysical evidence set the beginning of the opening phase in the Japan Sea basins, although a slightly early opening is assumed^81^. In this area, from $\sim$ 32 to $\sim$ 23 Ma, the volcanic front drifted towards the east, i.e., the Japan Sea started to open at the back of the Japanese island arc, showing active extension until 14–15 Ma^2,4,33,43^, times at which a neutral stress regime stage engaged with coexisting weak compressional and extensional stresses^33,67^ (Fig. 6). During this stage, the volcanic front migrated to the west^82^. In fact, reconstructions of the paleo-position of the volcanic front^2^, show short inwards (i.e., $\sim$ 100 km towards the west) migrations of the front itself. This behavior was interpreted as a progressive changing of the Pacific slab dip angle through time^2,4^. In the last few Ma ($< 5$ Ma) the northern Japan area is subject to compressional stresses, as shown by earthquake focal mechanisms and old extensional structure re-activated and inverted^2,31,67,69,83^. Indeed, from geological and geophysical evidence, we can currently observe that, in northern Japan, the hinge of the subducting slab is moving towards the upper plate, unlike other “westward” dipping subduction zones^18^.

### Kuril basin

The evolution of the Kuril basin nearly followed the Japan Sea one, being in active extension since $\sim$ 32 Ma until about 15-10 Ma, and seems now to be approaching to closure since Mid-Miocene^30,32^(Fig. 6).

Our work aim was mainly methodologic and was intended to evaluate the upper plate deformation regime in response to time-dependent convergence velocities and mantle flow. Our model shows that there are main compressional and extensional phases which are, to various degrees, observable howsoever in the basins which opened in north-eastern Eurasia through time (Fig. 6). In Figure 6, the deformation regimes observed from literature, e.g., geological and geophysical data, are shown. Of course, these data should be considered as a general overview, since the complexity of the evolution history of the area brought each region to undergo differential local stress regimes through time. From all collected data, appears evident that pure extension prevailed in the area until approximately 36–32 Ma and then compression started to affect the various regions. This first order behavior is attributed to velocity trend reversal of the subducting plate from lowering to rising. This relation between the deformation within the upper Eurasia plate and the convergence velocity of the Pacific oceanic plate is also found in literature^34,37^ and our results confirm this first order behavior.

In our models, thus, the timing of the evolution phases of the Kuril and Japan Sea back-arc basins is shifted earlier of about 15 Ma with respect to the observed stages, even though the duration of the main opening phase within the basin is coherent with what is observed and with the general life of a back-arc basin (i.e., $\sim$ 15 Ma^28^). On the contrary, it is more coherent with processes occurred in the Bohai Bay Basin, fitting even better the evolution of the East China Sea Shelf Basin. Furthermore, it is of course to mention that our 2D model does not take into account any local spatial variation in the 3rd dimension, e.g., the rotation of the Japan block, the interaction of the Eurasia plate with the North America (Okhotsk), Philippine Sea plates, the Bonin Arc and the India-Eurasia collision, which are extensively discussed in the literature, and seem to significantly affect the opening of both the Japan Sea and the Kuril Basins, but also the evolution of the East China Sea region^26,28,61,67,84–86^. Indeed, slab retreat is well known to be deeply influenced by the lateral changes of boundary conditions (e.g., the Mediterranean case^87^).

Neglecting the presence of the Philippine Sea adjacent subducting slab in our 2D models leads also to a predominant influence of the horizontal mantle flow on the Pacific slab dynamics. In fact, we can speculate that the Philippine Sea slab-related mantle counterflow could interact with first order mantle flow in the area, weakening its action, and leading to changes in timing with which the back-arc basins open and close in time. We also verified that the average velocity variations of the Pacific plate ($\sim$ 6.5 cm/a) in the last 57 Ma can be considered of the same order of magnitude of that of the Philippine Sea plate ($\sim$ 6.5 cm/a)^45,57^, so that our model could be considered as an average behavior of the entire margin under the tectonic influence of the Pacific subducting plate, at least until > 20 Ma. In fact, the Pacific plate alone subducted below the Eurasia plate until 20-10 Ma, when the Philippine Sea plate concurred to rise the geodynamic complexity of the area^88,89^. Thus, the influence of the Philippine Sea plate would need to be considered at least for the last $< 20$ Ma evolution history of the area. However, we remark that limitations related to the numerical models could influence both the timing of the whole subduction process, as well as its dynamics.

## Conclusions

Our work focuses on the influence of time-dependent velocity boundary conditions in geodynamic numerical modelling, and the related deformation in the upper plate of a subduction zone, in response to plate motion changes with time. This approach is coherent with evidence provided by plate tectonics: plates do not preserve constant velocity through long-term evolution.

With the inclusion of mantle flow, our numerical Model 2, shows that the position of the trench at subduction zones, is strongly affected by the variable kinematic evolution of the subducting plate. Along the Pacific-Eurasia plate boundary, the back-arc basin evolution experienced several phases of compression and extension, according to the trench movements and to increasing and decreasing of the subducting plate velocities.

This behavior is confirmed by geological and geophysical evidence along the entire north-eastern margin of the Eurasia plate, whose evolution history is strongly related to the Pacific plate subduction. However, being our work mainly methodologic, the phases of extension and compression resulted from our models should be considered as the main, first order, deformation behavior of the north-eastern Eurasia margin, that of course needs to be then integrated by regional tectonics of every basin that formed within the entire margin in the last 57 Ma.

On the contrary, Model 1, without mantle flow, shows no dip angle variation of the slab above 200 km depth and no back-arc basin opening throughout the entire subduction duration, which is inconsistent with the evolution of the entire geodynamic history of the eastern margin of the Eurasia plate.

Our work, thus, draw the attention on the importance of considering the interplay between plate kinematic variations and mantle flow, that primarily govern the geodynamic evolution of the eastern side of the Eurasian plate and, more in general, of subduction zones.

## Methods

Following^43^, we use a numerical setup in which a young Pacific plate (20 Ma) starts to subduct beneath Eurasia at 57 Ma, to bypass the Izanagi-Pacific spreading ridge subduction during the Paleocene. The age of the Pacific lithosphere then rapidly increases to reach 120 Ma at the right domain boundary, consistently with plate reconstruction models^24,57,89–91^. Although several authors examined the eastern margin of the Eurasia plate using numerical modelling tools, plate velocity variability according to plate motion reconstructions remains generally poorly investigated in geodynamic modelling^43,92–95^.

In this study, we use the numerical code I2VIS^96,97^, that was modified, following the approach described in the subsequent section, to include time-dependent velocity of the subducting plate as boundary conditions. It solves continuity, momentum, and energy equations using finite difference methods and combines the use of Lagrangian advecting points with a staggered Eulerian grid. Consequently, all the physical properties distributed on Lagrangian points are advected in agreement with the computed velocity field and then interpolated to the fixed Eulerian grid, i.e., the marker-in-cell technique^98^. Lithologies deform according to non-Newtonian viscous-plastic rheology. The ductile creep viscosity accounts for both dislocation and diffusion creep, as well as for Peierls creep at depths, and mineralogical phase changes are considered^99^.

Following^22^, simulations are performed using a grid resolution of 2111x351 nodes with variable grid spacing, giving a resolution which goes from 10 km at the domain margins to 1 km in the subduction area, in a 7000 km-wide and 1400 km-deep computational domain. The free surface upper boundary is simulated using the ’sticky air’ technique^96,100,101^.

Our model reproduces the subduction of a 5700 km-long oceanic plate under a 1300 km-long continental plate. Both continental and oceanic plates are composed of upper and lower crust, and lithospheric mantle. Periodic boundary conditions are implemented on the left and right sides of the domain^22,102^, whereas free-slip conditions are applied at the top and the bottom of the computational domain (Fig. S1, Supplementary Information). Velocity boundary conditions in the models were applied continuously at the oceanic lithospheric plate, within an area that goes from about 3500 to 6910 km along distance, so that only the oceanic plate is affected by them. Further details on the application of velocity boundary conditions are in the Supplementary Information (Tab. S1, Figs. S1, and S2, Supplementary Information).

### Kinematic and rheological constraints

In our numerical simulations changes in the Pacific plate motions with respect to a fixed Eurasia (Fig. 2) refer to the updated reconstruction models by^24^, with the inclusion of corrections to the Pacific rotations prior to 83 Ma^45,57^. Since the model is 2-dimensional, Pacific plate velocities have been calculated as an average along the profile in Figure 1 and, starting from 57 Ma to the present. They vary into an interval between 4 to 9 cm/a (Figs. 1, 2, 3 and 4, as well as Tab. S1 in the Supplementary Information). Variable slab velocities are imposed to the lithosphere (Supplementary Tab. S1), following results obtained by^24^. The process for implementing time-dependent velocities within the I2VIS numerical code relies on the application, to the oceanic plate velocity, of coefficients computed through four primary steps: (i) establishing key times for the transition of plate velocities; (ii) determining the time interval for which the plate velocity is constant; (iii) introducing a linear transitional change over a 1 Ma period before each key time, transitioning from the previous to the subsequent velocity change; (iv) keeping the constant plate velocity throughout the time interval specified in step (ii)^103–107^. Velocity changes are applied with the 1 Ma transition as shown also in Fig. 5a.

A sensitivity analysis on rheology of the models have been carried out (Tab. S3, Fig. S6, Supplementary Movies SM3 and SM4, in the Supplementary Information) to evaluate if plate stiffness and viscosity are affected by rapidly changing velocities. This analysis shows that the procedure with which we apply the velocity boundary conditions in the models does not affect the model rheology, not causing any deformation instability within the lithosphere, neither in the upper or lower subducting plates (Tab. S3, Fig. S6, and Movies SM3 and SM4, in the Supplementary Information), so that transition time of 1 Ma is acceptable.

Following the approach of^22^, we implemented a horizontal asthenospheric mantle flow, applied from 200 km depth, with a constant velocity of 3 cm/a, which corresponds to half of the average convergence velocity of the Pacific plate and is mainly aligned with predictions of the net rotation of the lithosphere with respect to the mantle^54,108,109^. Furthermore, a LVZ decoupling layer between a 100 and 200 km depth with a constant viscosity value of $10^{19}$ Pa s was included^22^. We used an average value for the thickness of the LVZ^52,53^, although it could have been slightly deviated from its average by tectonic processes occurred in the area until 57 Ma. Within this layer the presence of a small percentage of partial melt ($\sim \!1-2$%) and H_2_O causes a drop in the velocity of seismic waves and a low viscosity of the asthenosphere ($\sim \!10^{17}-10^{19}$ Pa s^46–49^) facilitating the relative motion of the lithosphere and the mantle.

### Supplementary Information

Supplementary Information 1.

Supplementary Movie S1.

Supplementary Movie S2.

Supplementary Movie S3.

Supplementary Movie S4.

## Data Availability

Numerical solutions were obtained using the I2VIS numerical code, which is not freely available, but can be provided upon email request sent to T. Gerya. The figures were made with the Generic Mapping Tools v. 6.0^110^, Python 3.9, and PyGMT^56^. The model input parameters, the used rheology, and the kinematic constraints, with setup allowing the model reproducibility, are provided in the Supplementary Information (e.g., Tabs. S1 and S2, Fig. S1, and Movies SM1-4), and in^98^.
