# Supplementary material for: Variable plate kinematics promotes changes in back-arc deformation regime along the north-eastern Eurasia plate boundary
Source: Sci Rep. 2024 Mar 27;14:7220. doi: 10.1038/s41598-024-57890-6 (PMC10973338; doi:10.1038/s41598-024-57890-6)
Supplement: Supplementary file 1 — Supplementary Information 1. [file 41598_2024_57890_MOESM1_ESM.pdf]

Supplementary Information

Variable plate kinematics promotes changes in back-arc deformation regime along the north-eastern Eurasia plate boundary

Eleonora Ficini[1], Marco Cuffaro[1], Carlo Doglioni[2,3], Taras Gerya[4]

1 CNR-IGAG, Rome, Italy
2 National Institute of Geophysics and Volcanology, Rome, Italy
3 Sapienza University of Rome, Department of Earth Sciences, Rome, Italy
4 ETH Zurich, Institute of Geophysics, Zurich, Switzerland

Corresponding author: Marco Cuffaro, marco.cuffaro@igag.cnr.it

Pacific plate velocities wrt Eurasia	
Time (Ma)	Velocity (cm/a)
57	5
55	7
53	6
52	5
47	6
39	4
32	5
26	6
25	7
19	8
9	9

Table S1. Kinematic evolution of the Pacific plate with respect to Eurasia fixed. List of time at which the velocity was changed in the models[1,2].

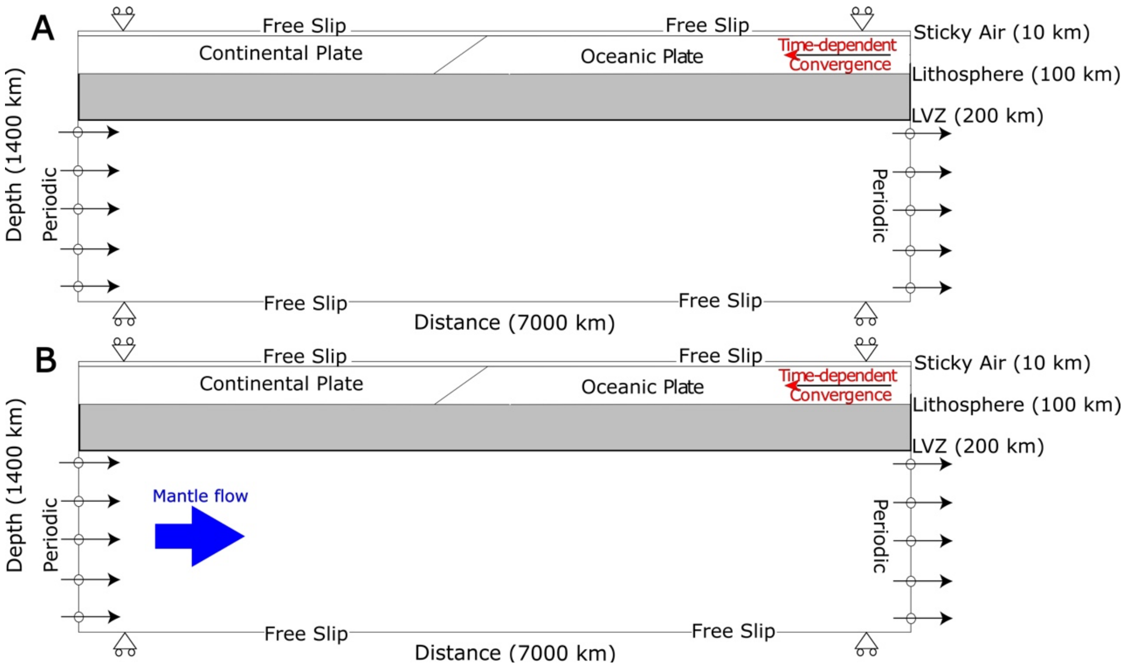

Figure S1. Model setups. Panel A represents setup for Model 1. The numerical domain includes time-dependent convergence velocities[1,2], a LVZ between 100 and 200 km depth, and no mantle flow. Panel B represents setup for Model 2, which includes time-dependent convergence velocities[1,2], a LVZ between 100 and 200 km depth, and a horizontal mantle flow applied with a constant velocity of 3 cm/a, which corresponds to half of the average convergence velocity of the Pacific plate[3-5]. Boundary conditions for top and bottom domain boundaries are free slip, whereas left and right domain boundaries are set to be periodic, to allow a throughgoing mantle flow, as in[6].

In red the direction of application of the subducting plate velocities, whereas in blue the direction of application of the mantle flow.

Rheological parameters									
**Material**	k $(W/m/K)$	H_r (W/m^3)	C_p (J/Kg)	sin (phi)	C (MPa)	**Flow law**	eta_0 $(Pa^n s)$	**n**	*lambda*
**Sediments**	$0.64+\frac{807}{T+77}$	1.5×10^{-6}	1000	0.150	1.0	Wet Qz.	1.97×10^{17}	2.3	1.0
**Upper cont. crust**	$0.64+\frac{807}{T+77}$	1.0×10^{-6}	1000	0.150	1.0	Wet Qz.	1.97×10^{17}	2.3	1.0
**Lower cont. crust**	$1.18+\frac{474}{T+77}$	0.25×10^{-6}	1000	0.150	1.0	Pl. (An75)	4.80×10^{22}	3.2	1.0
**Upper ocean. crust**	$0.64+\frac{807}{T+77}$	0.25×10^{-6}	1000	0.000	1.0	Wet Qz.	1.97×10^{17}	2.3	1.0
**Lower ocean. crust**	$1.18+\frac{474}{T+77}$	0.25×10^{-6}	1000	0.600	1.0	Pl. (An75)	4.80×10^{22}	3.2	1.0
**Low velocity layer**	$0.73+\frac{1293}{T+77}$	2.20×10^{-8}	1000	0.600	1.0	Dry Ol.	$3.98\times10^{18*}$	3.5	1.0
**Mantle**	$0.73+\frac{1293}{T+77}$	2.20×10^{-8}	1000	0.600	1.0	Dry Ol.	3.98×10^{16}	3.5	1.0
**Weak Zone**	$0.73+\frac{1293}{T+77}$	2.20×10^{-8}	1000	0.000	1.0	Wet Ol.	5.01×10^{20}	4.0	1.0

Table S2. Rheological parameters. Rheological[7] and thermal[8] parameters of materials used for the experiments.

Numerical models				
**Model name**	**Mantle flow**	**LVZ**	**Back-arc**	**Time-dependent kinematics**
**Model 1 (Reference)**	No	Yes	No	Yes
**Model 2**	Yes	Yes	Yes	Yes
**Model 3**	Yes	Yes	-	No
**Model 3a**	Yes	Yes	-	Yes
**Model 3b**	Yes	Yes	-	Yes

Table S3. Models overview. In this table, an overview of the models carried out for this study is shown. Model 1 and Model 2 are the main results of our work and are described in the main text (Figs. 3 and 4) and in this Supplementary Information (Figs. S2, S3 and S5). Whereas Model 3, which after 6 Ma splits in Model 3a and Model 3b, is described in this Supplementary Information (Figs. S6, Supplementary Movie SM3 and SM4) and represent the sensitivity analysis carried out to test the reaction of the oceanic and continental plates to changes of plate motions in the model. For models 3, 3a, and 3b, the opening of the back-arc basin in not evaluated since the short time of subduction evolution (~6-10 Ma).

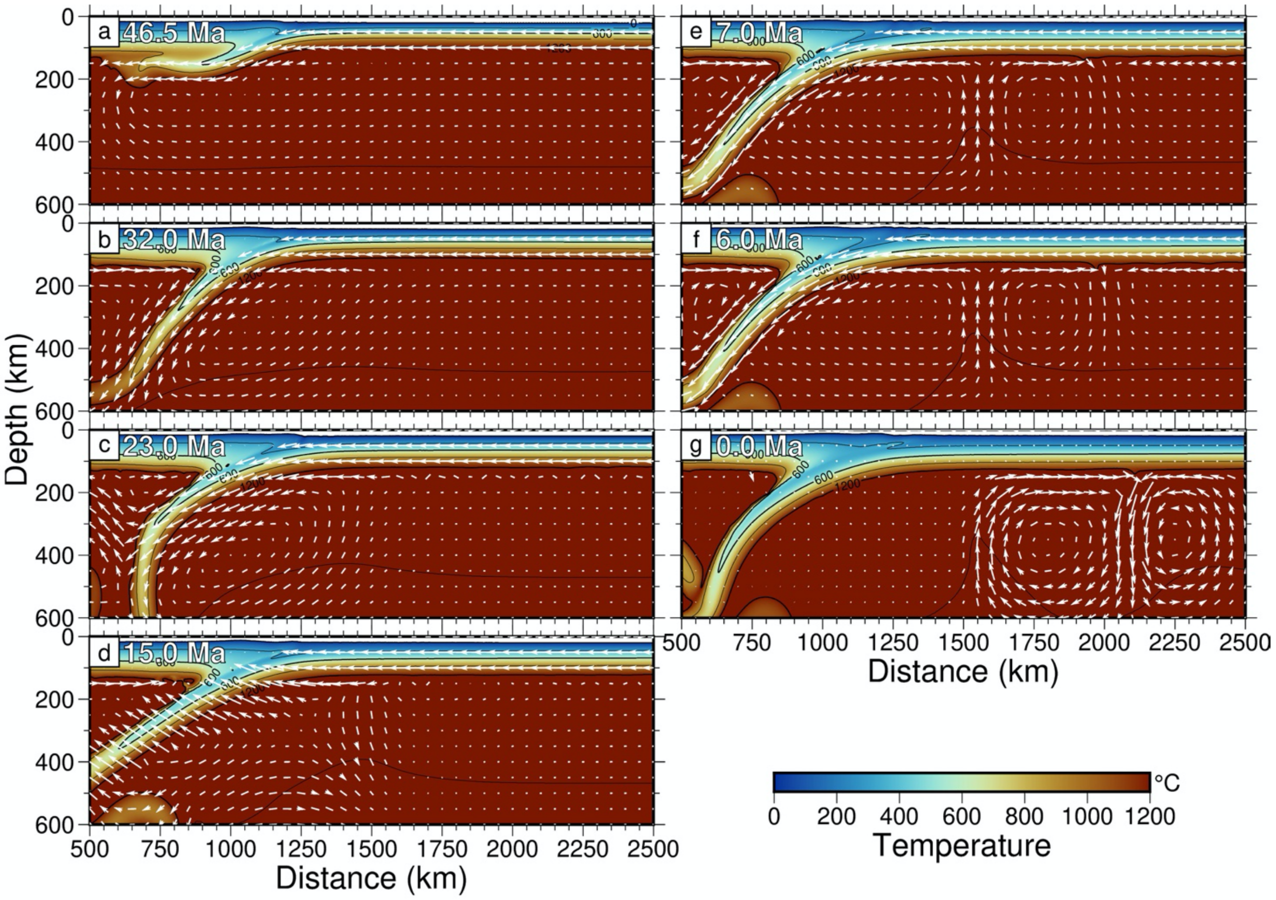

Figure S2. Results of models with LVZ and no mantle flow. These model results show the last 57 Ma subduction evolution of the Pacific plate under Eurasia. It includes time-dependent plate kinematics for the Pacific plate relative to Eurasia by updated model by[9], which include corrections to the Pacific rotations prior to 83 Ma[1,2]. These results show no back-arc basin opening throughout the entire subduction duration, which is inconsistent with the geodynamic history of the eastern margin of the Eurasia plate. From velocities, at depths, no counterflow within the mantle is generated to induce slab retreat, and eventually leading to back-arc basin opening.

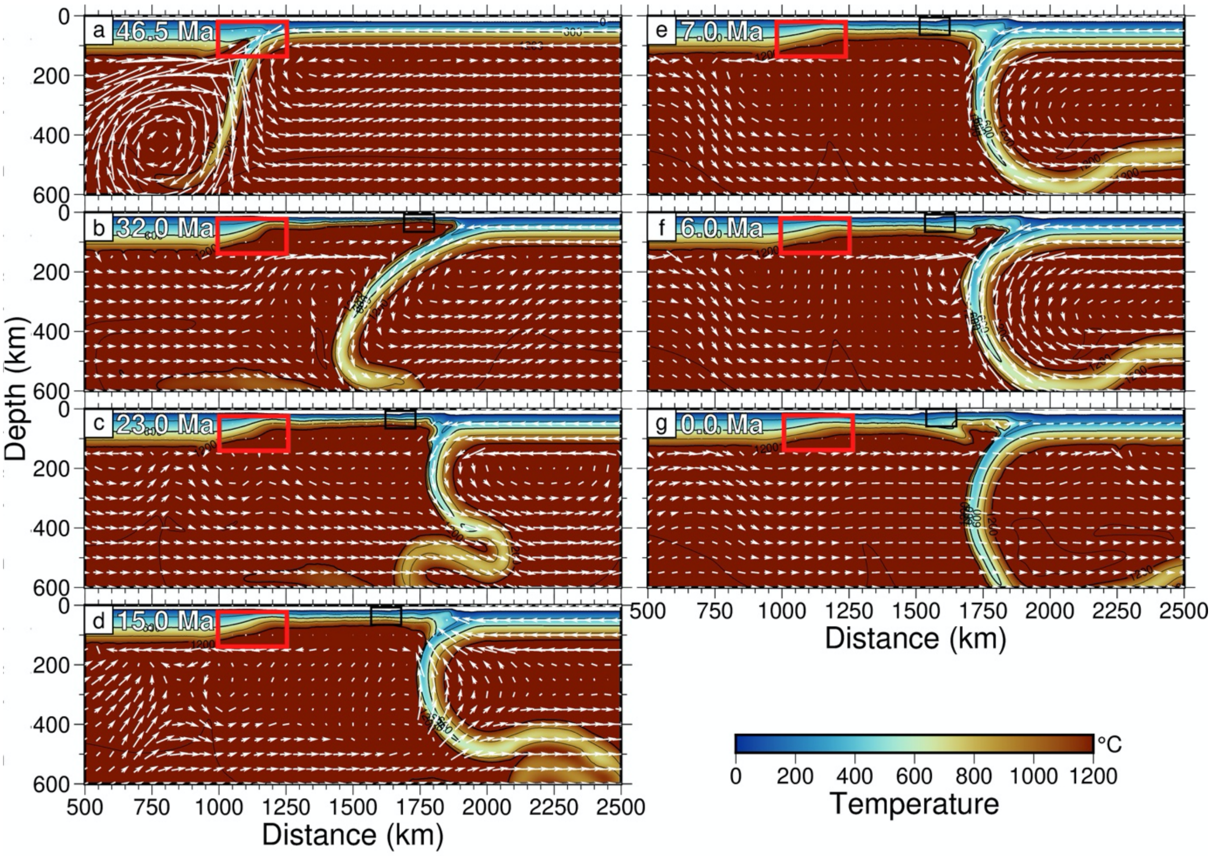

Figure S3. Results of models with LVZ and mantle flow. An extensional phase can be detected, beginning at ~ 46.5 Ma (panel a), with a developed back-arc already at ~ 32 Ma (panel b). At 32 Ma, a compressional phase starts (panel c), ended at ~ 23 Ma, when a new extensional phase begins. At ~ 15 Ma (panel d) a new inversion of the extensional trend starts. A very short opening trend starts at ~ 7 Ma (panel e), whereas at ~ 6 Ma a new inversion starts (panel f). Panel g shows the Present-day state of the north-eastern Eurasia margin. Velocities within the mantle show a slab which is entirely influenced by the eastward mantle flow in the initial opening phases of the back-arc basin, whereas it influences the slab mainly below 200 km in the subsequence phases. In fact, where the LVZ decollement level is located (100-200 km), the velocity field has no unique direction and shows, in the subduction wedge, several phases of westward and eastward directions. Moreover, a stagnation is observable at about the 660 km discontinuity. The black squares point to the area of lithospheric weakness which forms where the maximum thinning occurs, at 32 Ma, and from which the recent (i.e., 6.0 Ma) incipient subduction with opposite subduction polarity originates. The red squares point to the extended area in the continental upper plate.

Analysis of the horizontal deviatoric stress

As a comparison with Figure 5 in the main text, we computed the horizontal deviatoric stress (Sxx, Fig. S4) for Model 2, in a point situated at 350 km from the trench, and 25 km depth in the lithosphere, within the back-arc basin.

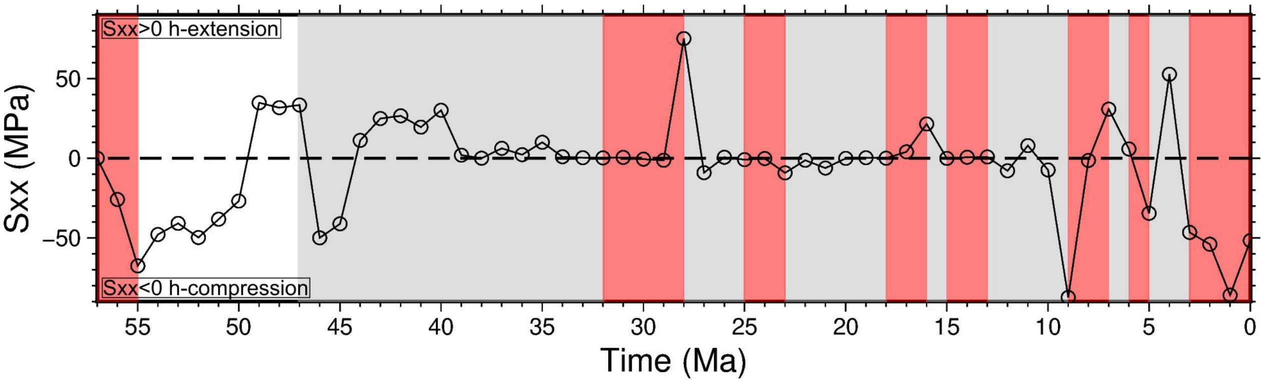

Figure S4. Evolution of the horizontal deviatoric stress (Sxx, black solid line, with circles) for Model 2. Sxx>0 represents horizontal extension (h-extension), whereas Sxx<0 represents horizontal compression (h-compression). The zero in the figure corresponds to the dashed black line, whereas red and grey areas correspond to trench advance and trench retreat phases, respectively, of Fig. 5 in the main text. The white area is for the stable trench location (stable TL, Fig. 5, main text).

Figure S4 shows that for the first 2 Ma compression occurred. At 55 Ma compression started to decrease and converted to extension at 49 Ma. From 47 Ma extension prevailed, which decreased at 39 Ma, whereas at 30 Ma some weak inversions started to occur. These latter became more effective in the last phases of the model run, from 10 to 0 Ma. This evolution of Sxx agrees with the general behavior of Model 2 (Figs. 4 and 5c in the main text), whereas the observed differences derive from local state of stress within the lithosphere, in the basin: in fact, for consistency we kept the measurement point at the same distance from the trench for all timesteps. However, the state of stress within the lithosphere at subduction zones changes with time and location, and is also related to other features such as, for example, the slab dynamics and its interactions with mantle transition zones[10, 11].

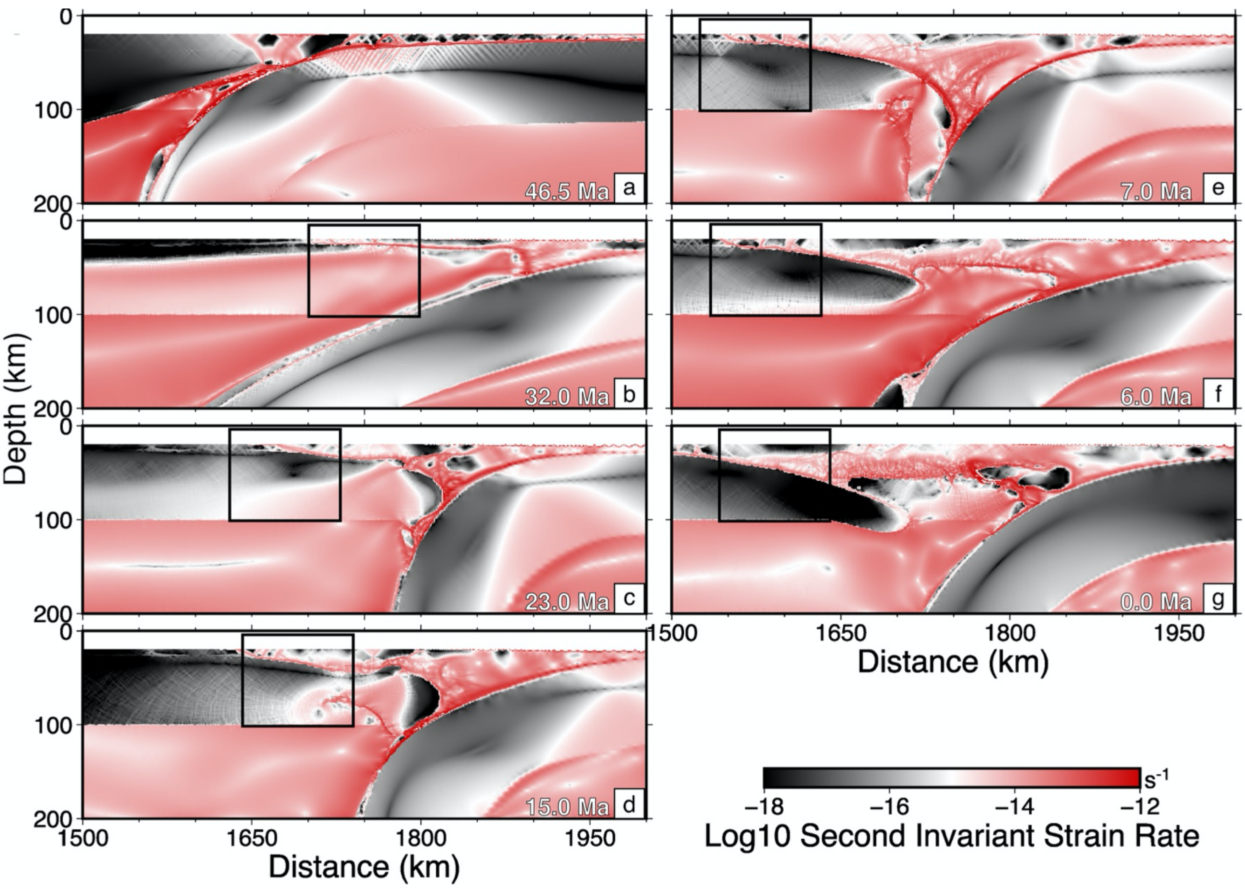

Figure S5. Log10 of the second invariant of the strain rate resulted from our numerical Model 2. Panel a shows the domain between 1000 and 1500 km. Every other panel shows the domain between 1500 and 2000 km. Basically, each panel shows the strain rate in the middle of extension (B, D, F) and compression (C, E, G) phases. Higher values of the second invariant in the trench area during the phases of the Pacific plate velocity increase (i.e., from 32 Ma) are shown. In this zoom on the trench area, the black squares represent the area of lithospheric weakness which forms where the maximum thinning occurs, at 32 Ma, and from which the recent (i.e., 6.0 Ma) incipient subduction with opposite subduction polarity originates.

Sensitivity analysis on transition between plate velocity changes

We made two simulations using the same setup as for Model 1 and Model 2, with parameters shown in Tabs. S2 and S3, to test the lithosphere stiffness and viscosity reaction to rapidly changing convergence velocities of the subducting oceanic lithospheric plate. Here, the subducting plate is pushed at constant 7 cm/a (i.e., average velocity) for the first 6 Ma, and in Figure S5 the Log10 of the Second invariant of the strain rate after the first 6 Ma of the Model 3 is reported. In Model 3, a constant velocity of the subducting oceanic plate is applied and, as it is shown in Figure S5, there are no indications of anomalous variation within the strain rate, nor in the upper or in the lower subducting plates. After 6 Ma, Model 3 splits in:

- Model 3a (Supplementary Movie SM3), in which the velocity change to 9 cm/a (i.e., maximum velocity) instantaneously occurs;

- Model 3b (Supplementary Movie SM4), in which the velocity change to 9 cm/a (i.e., maximum velocity) occurs with a transition of 1 Ma[12].

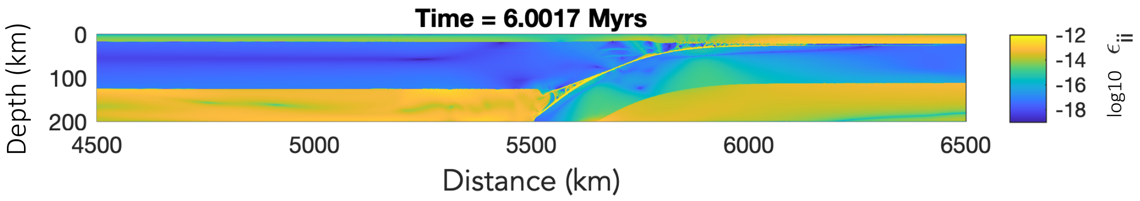

Figure S6. Model 3 Log10 of the second invariant of the strain rate, after the first 6 Ma with a constant plate velocity of 7 cm/a, just before the plate velocity change.

As it can be seen in the Supplementary Movies SM3 and SM4, the presence or not of a transition between changing velocities does not affect the model rheology, not causing any deformation peak within the lithosphere, neither in the upper or lower subducting plates. Thus, we chose to apply a transition of 1 Ma between two velocity changes in our Model 1 and Model 2, to agree with kinematic studies by[12].

Supplementary Movies

Supplementary_Movie_SM1. This movie shows Model 1 (with LVZ and without mantle flow) subduction evolution for the first 200 km depth. The colors represent the composition: light violet for the mantle, blue for lithospheric mantle, dark grey for the lower continental crust, light grey for the upper continental crust, light green for the gabbroic oceanic crust, dark green for the basaltic oceanic crust, orange and brown for sediments, and light blue for the weak hydrated zone which allows the subduction to start.

Supplementary_Movie_SM2. This movie shows Model 2 (with LVZ and with mantle flow) subduction evolution for the first 200 km depth. The colors represent the composition: light violet for the mantle, blue for lithospheric mantle, dark grey for the lower continental crust, light grey for the upper continental crust, light green for the gabbroic oceanic crust, dark green for the basaltic oceanic crust, orange and brown for sediments, and light blue for the weak hydrated zone which allows the subduction to start.

Supplementary_Movie_SM3. This movie shows Model 3a (with LVZ and with mantle flow) subduction evolution for the first 200 km depth, in which the velocity instantaneously changes from 7 cm/a to 9 cm/a (i.e., maximum velocity). Here the colors represent the Log10 of the second invariant of the strain rate.

Supplementary_Movie_SM4. This movie shows Model 3b (with LVZ and with mantle flow) subduction evolution for the first 200 km depth, in which the velocity changes from 7 cm/a to 9 cm/a (i.e., maximum velocity) with a transition of 1 Ma. Here the colors represent the Log10 of the second invariant of the strain rate.

References

1. Torsvik, T. et al. Pacific-panthalassic reconstructions: Overview, errata and the way forward. Geochem. Geophys. Geosystems 20, 3659–3689, DOI: 10.1029/2019GC008402 (2019).

2. Müller, R. et al. Gplates: Building a virtual earth through deep time. Geochem. Geophys. Geosystems 19, 2243–2261, DOI: 10.1029/2018GC007584 (2018).

3. Gripp, A. & Gordon, R. Young tracks of hotspots and current plate velocities. Geophys. J. Int. 150, 321–361, DOI:10.1046/j.1365-246X.2002.01627.x (2002).

4. Cuffaro, M. & Doglioni, C. Global kinematics in deep versus shallow hotspot reference frames. Special Pap. Geol. Soc. Am. 430, 359–374, DOI: 10.1130/2007.2430(18) (2007).

5. Doglioni, C. et al. Tectonically asymmetric earth: From net rotation to polarized westward drift of the lithosphere. Geosci. Front. 6, 401–418, DOI: 10.1016/j.gsf.2014.02.001 (2015).

6. Ficini, E., et al. Horizontal mantle flow controls subduction dynamics. Sci. Rep. 7, 7550, https://doi.org/10.1038/s41598-017-06551-y (2017).

7. Ranalli, G. Rheology of the Earth, *2nd ed. Chapman & Hall*, London (1995).

8. Clauser, C., Huenges, E. Thermal conductivity of rocks and minerals. In: Ahrens, T.J. (Ed.), Rock Physics and Phase Relations. AGU, AGU reference shelf 3, Washington DC, pp. 105–126 (1995).

9. Matthews, K. et al. Global plate boundary evolution and kinematics since the late paleozoic. Glob. Planet. Chang. 146, 226–250, DOI: 10.1016/j.gloplacha.2016.10.002 (2016).

10. Holt et al. Trench migration and overriding plate stress in dynamic subduction models. Geophys. J. Int. 201, 172–192, DOI: 10.1093/gji/ggv011 (2015).

11. Dasgupta et al. Controls of subducting slab dip and age on the extensional versus compressional deformation in the overriding plate. Tectonophysics 801, 228716 (2021).

12. Iaffaldano, G. A geodynamical view on the steadiness of geodetically derived rigid plate motions over geological time. Geochem. Geophys. Geosystems 15, 238–254, DOI: 10.1002/2013GC005088 (2014).
